# Antitumor and Immunoregulatory Activities of Seleno-β-Lactoglobulin on S180 Tumor-Bearing Mice

**DOI:** 10.3390/molecules23010046

**Published:** 2017-12-28

**Authors:** Su-jun Sun, Ying-ying Feng, Yan Zhang, Hai-yu Ji, Juan Yu, An-jun Liu

**Affiliations:** Key Laboratory of Food Nutrition and Safety, Ministry of Education, School of Food Engineering and Biotechnology, Tianjin University of Science and Technology, Tianjin 300457, China; jihaiyu1247@163.com (S.-j.S.); gfengyingying@foxmail.com (Y.-y.F.); haiyu11456@163.com (H.-y.J.); yujuan14615@163.com (J.Y.)

**Keywords:** seleno-β-lactoglobulin, S180 tumor-bearing mice, antitumor, immunoregulation

## Abstract

Degeneration of immune organs like thymus and spleen has been discovered in tumor-bearing mice; which increases the difficulties on oncotherapy. More effective drugs which target the protection of immune organs are expected to be researched. In this study; we aim to analyze the antitumor and immunoregulatory activities of seleno-β-lactoglobulin (Se-β-lg) on S180 tumor-bearing mice. Results indicated that Se-β-lg exhibited a remarkable inhibitory effect on S180 solid tumors with the inhibition rate of 48.38%; and protected the thymuses and spleens of S180-bearing mice. In addition, Se-β-lg could also balance the proportions of CD4^+^ and CD8^+^ T cells in spleens; thymuses and peripheral bloods; and improve Levels of IL-2; IFN-γ; TNF-α in mice serums. β-lg showed weaker bioactivities while SeO_2_ showed stronger toxicity on mice. Therefore our results demonstrated that Se-β-lg possessed stronger antitumor and immunoregulatory activities with lower side effects and had the potential to be a novel immunopotentiator and antitumor agent.

## 1. Introduction

Selenium is essential for the body due to the regulation on activity of seleno-enzyme-glutathione peroxidase [1,2]. Se plays critical roles in various physiological processes, which is associated with anticarcinogenesis, antioxidantion, immunoenhancement [3,4,5] and widely used as a dietary supplement, particularly [6,7,8,9]. Mao [10] found polysaccharide termed Se-GP11, extracted and purified from Se-enriched *Grifola frondosa* exhibited the antitumor effect through improving immunologic function of the tumor bearing mice. Yuan [11] had proved that Se-PFPs could significantly reduce tumors growth on nude mice bearing MDA-MB-231-derived xenograft tumors without toxic side effects.

β-Lactoglobulin (β-lg), the dominant whey protein in bovine milk, is an 18.4 kDa globular lipocalin protein that consists of 162 amino acid residues with a pI of approximately 5.4 [12,13,14]. β-lg is folded into a calyx formed by eight antiparallel b-strands and one a-helix at the outer surface of the b-barrel [15]. The unique structural character makes β-lg aggregate forming nanoparticles and when electrically charged, β-lg is able to bind various ligands and form electrostatic complexes [16]. β-lg nanoparticles have been applied for drug targeting such as beta casein nanoparticles for targeting gastric cancer and albumin conjugated nanoparticles for effective in vivo drug delivery across the blood-brain barrier [17].

In our previous study, a novel agent, seleno-β-lactoglobulin (Se-β-lg) was synthesized by introducing selenic acid groups to arginine and lysine’s primary amine in β-lg (applying for Chinese patent, No. 201410820824.3). The present study was designed to investigate the antitumor and immunoregulatory activities of Se-β-lg on S180 tumor-bearing mice.

## 2. Results and Discussion

### 2.1. Immune Organ Indexes and Tumor Inhibition Rate of Se-β-lg

As shown in Table 1, thymus index of tumor-bearing mice was significantly descended than that in normal group, while spleen index was obviously increased, which were consistent with previous finding [18,19], indicating that the proliferation of malignant neoplasm could damage the immune system severely by forming a micro-environment favorable for tumors growth, resulting in the splenomegaly and thymic atrophy. Results showed that Se-β-lg could significantly protect the immune organs in S180-bearing mice, while SeO_2_ exhibited serious toxic side effects due to the remarkable decrease compared with model group. The average tumor weight of model group was 2.79 ± 0.28 g. β-lg, SeO_2_ and three Se-β-lg groups showed remarkable inhibition effects on the growth of the S180 tumor compared to the model group with inhibitory rates of 21.51%, 53.05%, 20.79%, 45.88% and 48.38% respectively. These results indicated that SeO_2_ had strong killing effects of S180 cells in vivo with serious side effects, while Se-β-lg not only showed greater antitumor activity in S180-bearing mice than β-lg, but also possessed lower toxicity than SeO_2_.

### 2.2. T Lymphocyte Subpopulation of Thymus and Spleen

The two major subsets of T lymphocytes are defined by expression of CD4 or CD8 glycoproteins respectively [20]. CD8^+^ T cells play an important role in orchestrating the antitumor response by imparting cytolytic activity and cytokine expression. Tumor-bearing patients with massive infiltrated CD8^+^ T cells would have a better clinical outcome generally [21,22]. CD4^+^ T cells could recruit and activate innate immune cells such as natural killer cells and macrophages in anti-tumor immunity [23].

Results of proportions of T cell subsets in spleen were showed in Table 2. CD4^+^ and CD8^+^ T lymphocytes of tumor-bearing mice were remarkable decreased compared with normal mice. The proportion of T cells in SeO_2_ groups were even lower than that in model group, which indicated that SeO_2_ would promote the apoptosis of T cells in spleen and aggravate splenic injury, while β-lg and Se-β-lg could significantly balance the disorder of T lymphocytes, especially in high dosage of Se-β-lg group. The proportion of CD4^+^ T cells and CD8^+^ T cells reached 63.25% and 13.21% in Se-β-lg high dosage group, which indicated that Se-β-lg had the ability to effectively reduce the damage of malignant tumors.

As shown in Table 2, the proportions of T cell subsets in thymus were significantly increased in tumor bearing mice compared to normal group, maybe because the proliferation of tumor cells in vivo could accelerate the differentiation and proliferation of thymocytes as an immune response. Proportions of CD4^+^ and CD8^+^ T cells in medicine treatment groups were all decreased obviously compared with model group, particularly in Se-β-lg high dosage group with the proportion of CD4^+^ and CD8^+^ T cells being 20.21% and 16.23%. However, CD4^+^ T cell subsets in SeO_2_ group were 14.02% which were significant lower than that in normal group due to the toxic effects of SeO_2_ on both tumor cells and immune organs. Our results indicated that the Se-β-lg could effectively balance the population of T cells subsets in thymus and spleen of tumor-bearing mice, which would enhance the antitumor immunity.

### 2.3. T Lymphocyte Subpopulation of Tumor and Peripheral Blood

As shown in Table 3, after inoculation of tumor cells, the proportions of CD4^+^ and CD8^+^ T cells in peripheral blood of model group were reduced significantly compared to normal group, while only CD8^+^ T cells were found in solid tumors. After dealt with β-lg and Se-β-lg, the proportions of CD4^+^ and CD8^+^ T cells were significantly increased in peripheral blood, especially in H-Se-β-lg group, which indicated their ability of tumor inhibition effects by regulating T cells immune function. Compared to model group, CD4^+^ T cell and CD8^+^ T cell in peripheral blood were obviously decreased in SeO_2_ group, suggesting its cytotoxicity on body cells, considering the significant tumors inhibition effect. The results of proportions of CD8^+^ T cells in solid tumors with no significant intergroup difference indicated that the recognition capability of CD4^+^ T cells might exhibit greater antitumor effect.

### 2.4. Analysis of Cell Cycle Detection in Tumor Cells

As shown in Table 4, apoptosis ratio of tumor cells in drug treatment groups were significantly increased compared to model group with 13.21% in β-lg, 27.15% in SeO_2_, and 24.13% in Se-β-lg high-dosage group. The results showed that the effects of SeO_2_ and Se-β-lg on inducing tumors cells apoptosis were better than β-lg, which proved the remarkable inhibition effect of SeO_2_ on tumor cells in vivo, considering the immunosuppressive effect on immune system of tumor-bearing host SeO_2_ was not appropriate to clinical tumor therapy, while Se-β-lg exhibited better antitumor and immunoregulatory activities. As shown, an accumulation of cell numbers in G0/G1 phase in Se-β-lg groups was discovered dose-dependently, which indicated that Se-β-lg could inhibit S180 cells proliferation and promote tumors cells apoptosis by blocking cell cycle in G0/G1 phase in vivo.

### 2.5. Cytokine Detection

Interleukin-2 (IL-2), interferons-γ (IFN-γ) and tumor necrosis factor-α (TNF-α) are cytokines associated with stimulation and suppression of immune responses and play crucial roles in those pathological processes [24,25,26]. As shown in Table 5, the IL-2, IFN-γ and TNF-α levels in serums of model group were significantly decreased compared to normal group (*p* < 0.05), and those of SeO_2_ group were even lower than model group, suggesting its immunosuppression function on S180 tumor-bearing mice, which agreed with the data above. while the levels of cytokines in Se-β-lg were significantly higher than model group, which indicated that Se-β-lg exhibited a stronger immunoregulatory activity in balancing the levels of these cytokines in vivo with lower toxic side effects.

## 3. Materials and Methods

### 3.1. Animals and Cells

Kunming SPF mice (female, 8-week-old), body weight 18–22 g, were purchased from the Peking University Health Science Center (Beijing, China), and raised in a temperature and humidity controlled environment under a 12 h light/12 h dark cycle, and given food and water ad libitum. All mice were acclimatized to the facility for 1 week prior to experiments. All animal experiments were performed following the Animal (Control of Experiments) Ordinance of Laws of China.

Mouse sarcoma S180 cells were obtained from Immunological Laboratory of Tianjin Medical University and maintained in RPMI 1640 medium supplemented with 10% heat-inactivated fetal bovine serum at 37 °C in an atmosphere with 5% CO_2_ in 60 mm Petri dishe.

Mice were divided randomly into the following seven groups:

Group 1: Normal (N)
Group 2: Model (M)
Group 3: β-lg
Group 4: SeO_2_
Group 5: Low Se-β-lg (L-Se-β-lg)
Group 6: Middle Se-β-lg (M-Se-β-lg)
Group 7: High Se-β-lg (H-Se-β-lg)

Sarcoma 180 tumor cells (2 × 10^6^ cells/mouse) were inoculated in the right forelimb armpit of all mice in Group 2 to 7, and empty treatment was done in the control group. 15 days before and after tumor implantation, mice in groups 3–7 were administered, respectively the following 150 μg/kg lg, 100 μg/kg SeO_2_, 50 μg/kg Se-β-lg, 100 μg/kg Se-β-lg, 150 μg/kg Se-β-lg. Normal and model groups were both applied 0.2 mL/d saline solution orally throughout the experimental period. All mice were sacrificed and tumors, thymuses, spleens were cut and blood was collected from the retroocular venous plexus after 12 h fasting. All samples were stored at −80 °C immediately for biochemical analysis.

### 3.2. Thymus Index and Spleen Index

All the mice were killed by cervical dislocation and then spleen and thymus of the mice were excised and weighed on the balance at the end of the drug administration experiment. Index = average weight/average body weight.

### 3.3. Measurement of Tumor Weight and Tumor Inhibition Rate

Tumors were dissected after mice sacrificed and washed with serile saline and weighed precisely at the end of the experiment and calculate the anti-tumor rate.

Inhibitory rate/(%) = (the mean tumor weight of control group − the mean tumor weight of treated group)/the mean tumor weight of control group × 100.

### 3.4. PI Staining

The cell cycle distribution was analyzed by flow cytometry. After the mice sacrificed, tumor cells were harvested, washed twice with PBS and fixed in 70% ethanol, and stored at 4 °C for at least 18 h, washed twice again and then incubated with RNase 30 min at 37 °C, subsequently added propidium iodide (PI, BD Biosciences, San Jose, CA, USA) at 4 °C in the dark for 10 min. The stained cells were subjected to a BD FACSCalibur flow cytometer.

### 3.5. Detection of T Lymphocyte Subpopulation

CD4^+^ T cells and CD8^+^ T cells populations in thymus, tumor, spleen and blood was tested with PE-CD4^+^ and FITC-CD8^+^ antibodies. Thymocyte suspensions were produced by forcing the samples through a wire-mesh screen and incubated in RBC lysis buffer for 15 min to lyse red blood cells, then diluted with PBS. Following centrifugation (1500 rpm, 5 min), lymphocytes were detected and then incubated with the antibodies for 30 min at 37 °C in the dark. Cells were analyzed after PBS washing on a flow cytometer and analyzed with CELLQuest software (BD Biosciences, San Jose, CA, USA).

### 3.6. Detection of IL-2, IFN-γ and TNF-α in Peripheral Blood

IL-2, IFN-γ and TNF-α in serum of each group were assessed by ELISA Kit (Hufeng Biotech. Co., Shanghai, China) under the manufacturer’s instruction. Then the plate was read on a BioRad Model 680 Microplate Reader at 450 nm.

### 3.7. Statistics Analysis

All experiments data are expressed as means ± SD. One-way analysis of variance (ANOVA) followed by the S–N–K test was used to determine the differences between groups under different conditions. *p*-Value less than 0.05 was considered statistically significant.

## 4. Conclusions

Se-β-lg exhibited a strong antitumor activity on S180 tumor-bearing mice in vivo, which could effectively protect the thymus and spleen from solid tumors, balance the proportions of T cells in peripheral blood, induce the apoptosis of S180 tumor cells via blocking cell cycle in G0/G1 phase and enhance the secretion of tumor-related cytokines. Our results indicated that Se-β-lg processes a better immunoenhancement effect than β-lg and a lower toxic effect than SeO_2_, which would provide a theoretical basis on its practical applications in food and drug industries.

## Figures and Tables

**Table 1:** Immune organ indexes and tumor inhibition rate of Se-β-lg.

Treatment	Dose	Thymus Index	Spleen Index	Tumor Weight	Inhibitory Rate
	(μg/kg)	(mg/g)	(mg/g)	(g)	(%)
N	-	3.21 ± 0.36	4.89 ± 0.31	-	-
M	-	1.63 ± 0.14 ^a^	8.16 ± 0.21 ^a^	2.79 ± 0.28	-
β-lg	150	1.98 ± 0.14 ^b^	6.47 ± 0.31 ^b^	2.19 ± 0.12 ^b^	21.51
SeO_2_	100	1.33 ± 0.11 ^b^	3.17 ± 0.13 ^b^	1.31 ± 0.11 ^b^	53.05
L-Se-β-lg	50	1.94 ± 0.12 ^b^	6.48 ± 0.41 ^b^	2.21 ± 0.19 ^b^	20.79
M-Se-β-lg	100	2.76 ± 0.28 ^b^	5.38 ± 0.42 ^b^	1.51 ± 0.10 ^b^	45.88
H-Se-β-lg	150	3.18 ± 0.36 ^b^	4.72 ± 0.21 ^b^	1.44 ± 0.12 ^b^	48.38

Note: ^a^ *p* < 0.05 compared to normal group; ^b^ *p* < 0.05 compared to model group.

**Table 2:** T lymphocyte subpopulation in spleen and thymus.

Treatment	Spleen		Thymus	
	CD4/(%)	CD8/(%)	CD4/(%)	CD8/(%)
N	67.01 ± 6.19	15.13 ± 1.21	17.07 ± 1.19	1 1.21 ± 1.23
M	50.21 ± 3.25 ^a^	8.52 ± 0.88 ^a^	28.21 ± 2.24 ^a^	21.52 ± 1.88 ^a^
β-lg	54.36 ± 4.12 ^b^	11.22 ± 1.73 ^b^	24.89 ± 2.12 ^b^	19.22 ± 1.73 ^b^
SeO_2_	44.02 ± 3.07 ^b^	6.22 ± 0.82 ^b^	14.02 ± 1.07 ^b^	13.15 ± 1.22 ^b^
L-Se-β-lg	53.98 ± 4.16	12.44 ± 1.12 ^b^	23.98 ± 2.23 ^b^	21.44 ± 2.16
M-Se-β-lg	61.68 ± 3.41 ^b^	13.27 ± 1.14 ^b^	21.68 ± 2.41 ^b^	17.28 ± 1.23 ^b^
H-Se-β-lg	63.25 ± 5.25 ^b^	13.21 ± 1.19 ^b^	20.31 ± 2.25 ^b^	16.23 ± 1.48 ^b^

Note: ^a^ *p* < 0.05 compared to normal group; ^b^ *p* < 0.05 compared to model group.

**Table 3:** T lymphocyte subpopulation of peripheral blood and tumor.

Treatment	Peripheral Blood		Solid Tumor	
	CD4/(%)	CD8/(%)	CD4/(%)	CD8/(%)
N	42.22 ± 3.17	23.01 ± 2.19	-	-
M	28.63 ± 2.11 ^a^	18.52 ± 1.12 ^a^	0.09 ± 0.003	41.52 ± 3.12
β-lg	32.79 ± 3.25 ^b^	19.88 ± 1.14 ^b^	0.12 ± 0.003	40.24 ± 3.11
SeO_2_	24.02 ± 2.11 ^b^	16.04 ± 1.12 ^b^	0.14 ± 0.002	38.41 ± 2.21
L-Se-β-lg	31.98 ± 2.20 ^b^	21.11 ± 1.16 ^b^	0.12 ± 0.004	39.87 ± 3.29
M-Se-β-lg	38.68 ± 3.21 ^b^	21.28 ± 2.19 ^b^	0.13 ± 0.003	41.01 ± 2.41
H-Se-β-lg	44.20 ± 3.24 ^b^	23.19 ± 1.27 ^b^	0.11 ± 0.004	38.82 ± 3.34

Note: ^a^ *p* < 0.05 compared to normal group; ^b^ *p* < 0.05 compared to model group.

**Table 4:** Distributions of cell cycle and apoptosis of tumor cell.

Groups	G0/G1/(%)	S/(%)	G2/M/(%)	Apoptosis/(%)
M	31.44 ± 2.35	54.51 ± 3.12	14.52 ± 1.63	2.24 ± 0.32
β-lg	42.32 ± 2.32 ^b^	44.63 ± 3.42 ^b^	14.34 ± 1.24	13.21 ± 1.05 ^b^
SeO_2_	50.21 ± 3.32 ^b^	34.32 ± 2.15 ^b^	15.26 ± 1.22	27.15 ± 2.53 ^b^
L-Se-β-lg	38.13 ± 2.46 ^b^	48.27 ± 4.13 ^b^	14.47 ± 1.45	11.32 ± 1.03 ^b^
M-Se-β-lg	43.23 ± 2.12 ^b^	41.32 ± 3.23 ^b^	13.51 ± 1.63	18.13 ± 1.24 ^b^
H-Se-β-lg	49.11 ± 3.43 ^b^	36.92 ± 3.05 ^b^	15.37 ± 1.35	24.13 ± 2.12 ^b^

Note: ^b^ *p* < 0.05 compared to model group.

**Table 5:** Results of IL-2, IFN-γ, and TNF-α levels in serums of mice.

Treatment	IL-2 (pg/mL)	IFN-γ (ng/L)	TNF-α (ng/L)
N	4.01 ± 0.19	119.83 ± 7.01	117.89 ± 4.07
M	2.52 ± 0.11 ^a^	112.34 ± 5.93 ^a^	91.09 ± 5.99 ^a^
β-lg	2.89 ± 0.15 ^b^	116.71 ± 4.97	97.77 ± 7.03
SeO_2_	2.02 ± 0.11 ^b^	100.84 ± 4.93	80.90 ± 4.99
L-Se-β-lg	2.98 ± 0.16 ^b^	118.8 ± 5.98	101.86 ± 4.04 ^b^
M-Se-β-lg	3.68 ± 0.21 ^b^	124.5 ± 8.03 ^b^	111.56 ± 3.09 ^b^
H-Se-β-lg	3.81 ± 0.25 ^b^	133.03 ± 7.07 ^b^	120.09 ± 6.13 ^b^

Note: ^a^ *p* < 0.05 compared to normal group; ^b^ *p* < 0.05 compared to model group.
